# Genome-Wide Analysis of 24-nt siRNAs Dynamic Variations during Rice Superior and Inferior Grain Filling

**DOI:** 10.1371/journal.pone.0061029

**Published:** 2013-04-12

**Authors:** Ting Peng, Yanxiu Du, Jing Zhang, Junzhou Li, Yanxia Liu, Yafan Zhao, Hongzheng Sun, Quanzhi Zhao

**Affiliations:** Henan Engineering Laboratory for Rice and Key Laboratory of Physiology, Ecology and Genetics Improvement of Food Crop in Henan Province, Henan Agricultural University, Zhengzhou, China; Rush University Medical Center, United States of America

## Abstract

24 nt-siRNAs are the most abundant small interfering RNAs in rice grains aside from microRNAs. To investigate the roles that 24 nt-siRNAs played in the poor grain filling of rice inferior grains, dynamic variations of 24 nt-siRNAs in inferior grains were compared with those of superior grains by using small RNA deep sequencing technology. The results showed that 24 nt-siRNAs derived from multiple regions of rice genome, and the maintenance of the two strands of 24 nt-siRNA duplex was a non-random process. The amounts of 24 nt-siRNAs declined with the process of grain filling in both superior and inferior grains, but 24 nt-siRNAs in inferior grains was much higher than that of superior grains in each period we sampled. Bioinformatics prediction indicated that 24 nt-siRNAs targeted on more genes involved in most of the known KEGG rice pathways, such as the starch and sucrose biosynthesis pathway. Combined with digital gene expression profiling of target genes, 24 nt-siRNAs mapped on the antisense strands of exons were specifically investigated, but the abundance of 24 nt-siRNAs did not show negative correlations with their corresponding target genes. The results indicated that 24 nt-siRNAs were not involved in down-regulation of target genes. The potential biological meanings for this inconsistency were probably the results of methylation directed gene expression activation, or competition for small RNA stability methylation.

## Introduction

Grain filling is a major characteristic that influences the quality and yield of rice grains. The filling rate difference between cultivars is controlled by qualitative trait loci (QTLs) [1]. While, aside from genotype reasons, grain filling rates in the same cultivar and the same panicle vary due to the position of grains on the panicle. In the architecture of rice panicle, the earlier flowered spikelets on apical primary rachis branches are defined as superior spikelets, from which grains fill faster and are heavier. In contrast, the later flowered spikelets on lower secondary rachis branches are defined as inferior spikelets, which have a slower grain filling rate and produce poor quality grains [2], [3]. It was found that poor grain filling of inferior spikelets was a key factor limiting the yield potential of current rice varieties [4]. Thus, investigation on the mechanism of grain filling is essential to improve rice yield for the production of “super” high-yield rice cultivars that have numerous spikelets on the panicle to provide sufficient staple food for more than half of the world’s population.

Previous studies investigated the physiological difference of auxins and enzyme activities between the superior and inferior grains, and found a higher enzyme activity and auxin concentration in superior spiklets [5], [6]. Researches based on gene expression profile and protein 2-D electrophoresis profile also found genes or proteins expressed differentially between the two kinds of tissues [4], [7]. But how those genes are regulated is still an elusive question to investigators.

In recent years, plant small RNAs are found to regulate genes involved in plant development, maintenance of genome integrity, and biotic and abiotic response [8]–[10]. Of these small RNAs, two kinds of them are found abundant in plants: microRNA (miRNA) and small interfering RNA (siRNA) [11]. miRNAs derive from single-stranded RNA precursors which form a hairpin structure [12], while siRNAs are generated from long double-strand RNA [13]. Both of them can cause translational inhibition or cleavage of target mRNAs [14], [15]. In rice grains, large amounts of small RNAs were detected by using deep sequencing technology [16]–[18]. Peng *et al.* (2011) studied the expressional difference of miRNAs between superior and inferior grains and found that differentially expressed miRNAs might participate in regulating hormone metabolism, carbohydrate metabolic pathways, and cell division during rice grain development. However, numerous siRNAs were also identified in rice grain development. Previous investigations suggest that endogenous siRNAs were also important in regulation of gene expressions [9], [11], [14], [19]. In this study, we focused on the most abundant siRNAs of 24-nt in length in rice grain development to investigate their dynamic variations and the regulation of their target genes during grain filling process. The putative functions of 24 nt-siRNAs during grain filling were discussed.

## Results

### Analysis of siRNA Reads from Deep Sequencing

Small RNA deep sequencing of the developing grains resulted in a total of 131,888,453 clean reads from the 10 sample libraries, varying from 10,658,388 to 17,702,636 reads in each library. In the small RNA population, 12,148,038 were identified as siRNAs that have predicted perfect match duplex and two bases 3′-overhanging structure, representing 765,268 unique siRNA sequences (Table 1). About one fourth of these siRNA sequences could not be mapped on the japonica rice reference genome (Rice Genome Annotation Project, release 6.1), which might come from sequencing error or single nucleotide polymorphisms (SNPs) in the genotype we used. In order to increase the reliability of the analysis, the siRNAs were further filtered to eliminate sequences that could not be mapped to the reference genome. As a result, 604,868 unique siRNA sequences were retained for further analysis. Since siRNAs were generated from double strand RNAs, two siRNA duplex strands should be from the same genomic loci. Nevertheless, 192,313 of these retained siRNAs were mapped to more than one position on the reference genome, which may generate false identification due to genome complexity, and thus removed. Finally, 412,555 unique siRNA sequences with unambiguous positions on the genome and characteristic of double strand duplex structure were used for subsequent analyses (Table 1).

**Table 1:** Summary of small RNA numbers in the ten libraries.

	S_10DAF	S_15DAF	S_21DAF	S_27DAF	S_35DAF	I_10DAF	I_15DAF	I_21DAF	I_27DAF	I_35DAF
**Number of total clean reads**	14,248,902	13,297,350	11,378,685	11,647,614	10,658,388	11,630,087	11,722,844	15,774,443	17,702,636	13,827,504
**Number of mapped reads**	12,779,360	11,811,091	9,941,669	9,867,610	8,789,166	10,621,487	10,754,320	14,302,943	15,851,871	12,122,729
**Reads number of siRNAs with duplex structure**	1,277,501	845,336	473,392	668,230	731,697	2,129,858	1,825,379	1,770,892	1,641,681	784,072
**Reads number of siRNAs** **unambiguously mapped**	816,706	433,010	147,284	131,530	100,723	1,724,709	1,483,629	1,353,252	1,106,900	347,937
**Reads number of 24 nt-siRNAs**	738,515	368,377	93,525	68,383	56,503	1,650,145	1,430,126	1,278,948	1,056,603	288,764
**Number of unique 24 nt-siRNAs**	84,853	65,744	26,949	23,028	18,499	78,896	88,969	91,319	102,123	48,904

**Figure 1:**
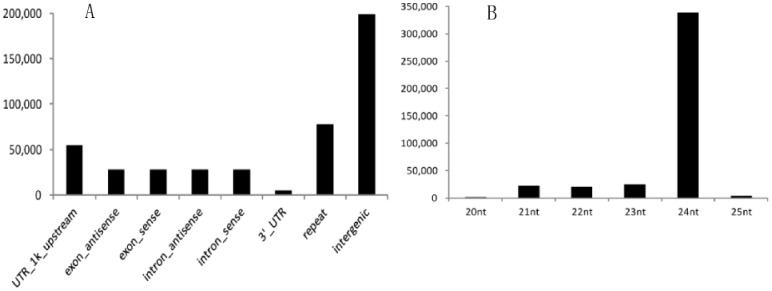
Localization and length distribution of unambigously mapped siRNAs in rice grains. A, location distribution; B, length distribution. The represents axis indicates the numbers of siRNA in each category.

The resulting siRNAs were mainly from 5′-UTR and 3′-UTR, exons, introns, repeat and intergenic regions of the genome (Figure 1A), indicating multiple sources of siRNA biogenesis. Of these unambiguous mapped siRNAs, their lengths varied from 20 nt to 25 nt. Strikingly, of these siRNAs, more than 80% are 24-nt siRNAs (Figure 1B). We analyzed the nucleotide compositions of each 24 nt-siRNA and found an “A” preferred bias on the first position at the 5′ end and a corresponding “U” bias on the 22nd position at the 3′ end (Figure S1).

**Figure 2:**
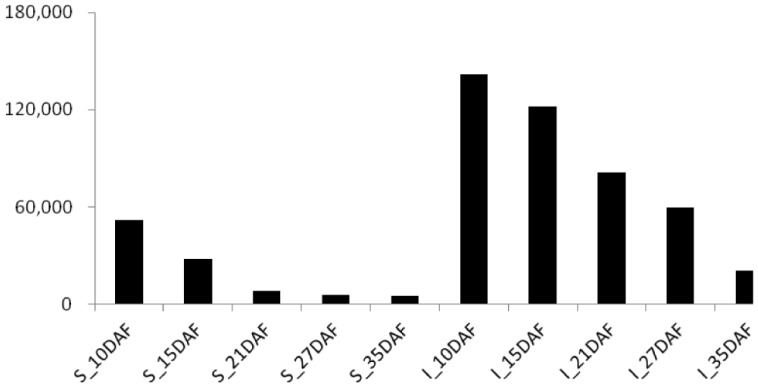
Dynamic variation of 24 nt-siRNAs in superior and inferior grains. The horizontal axis represents sample periods of superior and inferior grains, and the vertical axis represents number (in TPM) of 24 nt-siRNAs in each library.

The expression dynamics of the 24-nt siRNAs showed a declining trend during the process of grain filling, but the inferior grains contained more 24 nt-siRNAs than superior grains in each period we sampled. The superior grains presented 738,515 24 nt-siRNAs at 10 days after fertilization (DAF) and declined to 56,503 at 35DAF stage. In contrast, the inferior grains had much abundant 24-nt siRNAs with a similar trend declining from 1,650,145 at 10DAF stage to 288,764 at 35DAF stage (Figure 2 and Table 1). Four 24 nt-siRNAs were chosen to validated the robustness of the small RNA sequencing data by qRT-PCR analysis, and the results were in accordance with the sequencing results (Figure S2).

### Asymmetric Retention of siRNAs and siRNA*s

**Figure 3:**
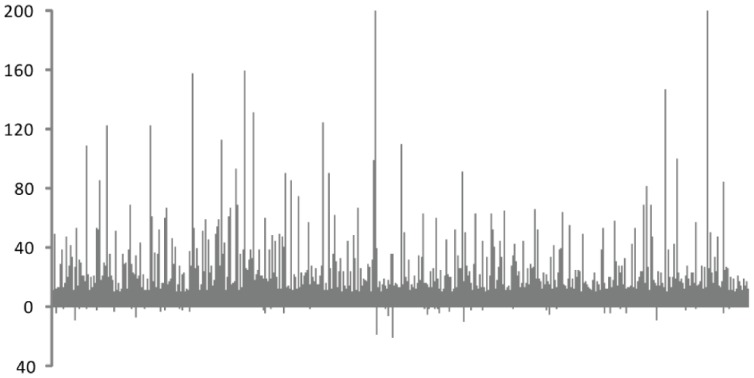
Frequency of 24 nt-siRNAs and 24 nt-siRNA*s. The horizontal axis represents 24 nt-siRNA pairs. The vertical axis represents frequency in TPM. Bars above and below the horizontal axis represent frequency of 24 nt-siRNA and 24 nt-siRNA*, respectively.

In miRNAs, after the pre-miRNA precursor hairpin structure were cleaved, mature miRNAs were released and the miRNA*s were degenerated in most circumstance [20]. In this study, siRNAs with higher expressions were designated as siRNAs and their complement strands were designated siRNA*s. In our datasets, we found the fate of the two duplex strand siRNAs were quite different, especially high frequency siRNAs greater than 10 transcripts per million (TPM). In the small RNA library of 10DAF, 765 pairs of 24 nt-siRNA were detected with the siRNA expression level greater than 10 TPM, but most of their corresponding siRNA*s were detected at very low frequency (Figure 3 and Table S1). Because the 24 nt-siRNAs in this analysis were unambiguously mapped to unique position on the genome, the asymmetric frequency of siRNAs and siRNA*s were not likely a false positive result. This indicated that the retention of the two duplex strands of siRNAs was also selective, like that of miRNAs.

### 24 nt-siRNAs Expressed in a Stage Specific Way

**Figure 4:**
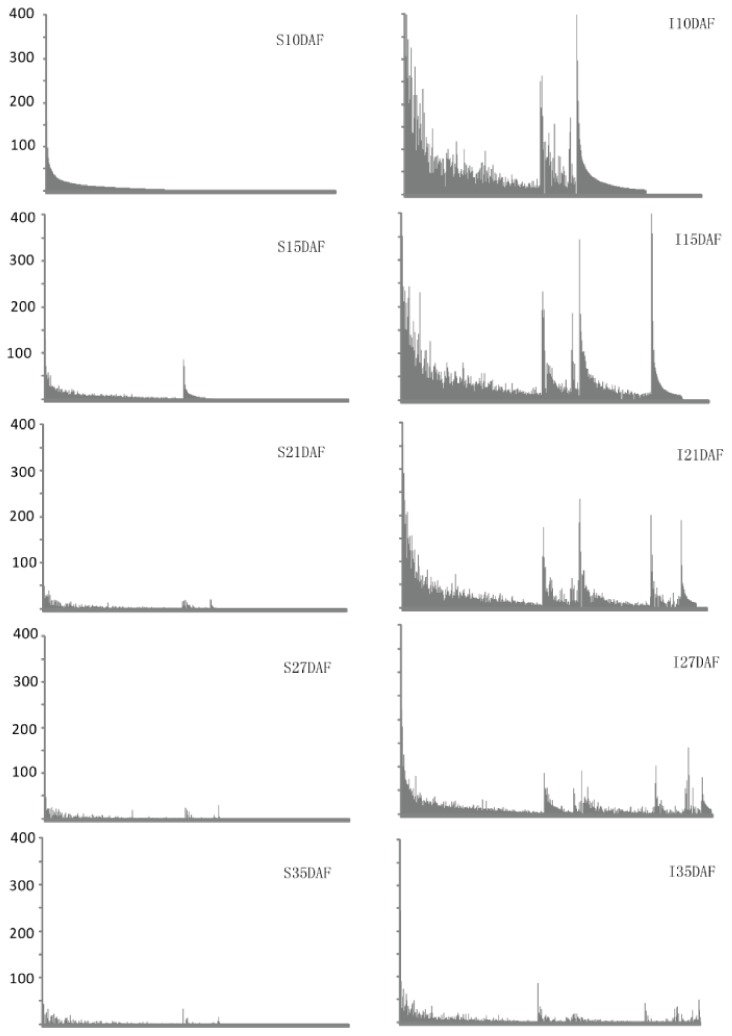
Frequency of 24 nt-siRNAs greater than 10TPM in the superior and inferior grains. The horizontal axis indicates 24 nt-siRNA, and vertical axis indicates frequency in TPM. The siRNAs were arranged in the same order for the sake of comparison between superior and inferior grains of different stages. S indicates superior grain and I indicates inferior grain. DAF represents day after flowering.

4215 unambiguously mapped 24 nt-siRNAs were detected at a frequency of greater than 10 TPM at least in one of the ten libraries. Of these siRNAs, 1775 were only detected in inferior grains, and most of these siRNAs were detected in one or specific stage during grain filling (Table S2). It was also observed that in both superior and inferior grains, the number of 24-nt siRNAs showed a declining trend during the filling stage. However, compared with superior grains, the inferior grains contain much more 24-nt siRNAs in each of the five stages we sampled (Figure 4). For example, siR95400, siR290778, siR326529 and siR326225 were all 24 nt-siRNAs with frequency greater than 10 TPM, which were mapped on the anti-sense strand of LOC_Os12g40000 exon1. siR95400, siR290778 and siR326529 were only detected in inferior grains. siR326225 was detected in both superior and inferior grains, but showed much higher frequency in inferior grains in each of the five period libraries (Table S2).

### Key Enzymes Involved in Processing 24 nt-siRNAs Expressed Higher in Inferior Grains

The biogenesis of 24 nt-siRNAs require DNA-dependant RNA polymerase IV, RNA-dependant RNA polymerase (RDR2), and DCL3 [21]. In digital gene expression profiling of rice superior and inferior grains, expression of three PolIV subunit genes, RDR2 and two DCL3 genes were detected, and all these genes were expressed higher in inferior grains (Table S3 and Figure S3), which explained the more abundant 24 nt-siRNAs in inferior grains. Interestingly, rice has two copies of DCL3. It was reported that the processing of 24 nt phased small RNAs requires OsDCL3b rather than OsDCL3a [22]. In our digital gene expression profile data, OsDCL3b was expressed at negligible level, and the OsDCL3a was found expressed much higher than OsDCL3b in each period in both superior and inferior grains. The result was further proven by data from Rice Genome Annotation Project rice gene expression database (Figure S4), which showed that DCL3b was expressed higher in pre-emergence inflorescence and very low in other sampled periods. This might be the result of functional diversification between duplicated OsDCL3 genes, with each copy expressed in specific period.

### siRNA Target Prediction and KEGG Analysis of Target Genes

24 nt-siRNAs greater than 10 TPM were subjected to target prediction against all the cDNA sequences in rice (RGAP, release 6.1). 3594 of the 4215 siRNAs were predicted to target on 14196 sites, representing 8270 genes, with most siRNAs targeting on multiple sites. In rice, there are 116 known pathways in KEGG database (http://www.genome.jp/). KEGG analysis indicated that these predicted target genes were involved in 106 of the 116 known pathways in rice (Table S4). The 24 nt-siRNAs and their target genes were investigated individually in each stage sample. As demonstrated in Table S4, 24 nt-siRNAs targeted more genes in each of the stage of the inferior grains than that in superior grains. This trend was observed in most of the 106 pathways involved, and the differences of 75 pathways between five stages of superior and inferior grains were significant as indicated by pair-wise T test (Table S4).

Starch is the primary and main component of rice grains. Therefore, the starch and sucrose pathway genes were investigated in detail. Of the 4215 24 nt-siRNAs with frequency greater than 10 TPM, 138 were predicted to target genes related with starch and sucrose pathway. In superior grains of 10DAF, 23 genes in the starch and sucrose pathway were predicted to be the target of siRNAs. Whareas, in inferior grains at 10DAF, 99 target genes were predicted. This result was consistent with previous studies that the inferior grains had lower expression level of starch and sucrose synthesis genes [7].

### Correlation between 24 nt-siRNAs and their Target Genes Revealed by Digital Gene Expression Profile

The expression of predicted 24 nt-siRNA target genes was validated by digital gene expression profile by using deep sequencing technology. Of the 8270 predicted target genes, 4,114 were detected as expressed in at least one of the ten libraries. Frequency of 24 nt-siRNAs were linked with their corresponding predicted target genes in 5,538 pairs. It was found that in superior grains, 436 siRNAs and their target genes were significantly positively correlated as indicated by pearson correlation index, and only eight pairs were significantly negative. While, in inferior grains, 636 siRNAs were significantly positively correlated with their corresponding target genes, and 54 pairs were significantly negative (Table S5). To avoid the possibility of false positive prediction of the target genes, 38 siRNAs which could be perfectly mathched on the anti-sense strand of cDNA transcripts were chosen and subjected to pearson correlation analysis. The results showed that in superior grain, none was detected as significantly negatively correlated and only five pairs were significantly positive. In inferior grain, only three 24 nt-siRNAs and their target genes were significantly positive, and no pairs was significantly negative. These results indicate that the 24 nt-siRNAs are probably not involved in the down regulation of the target genes (Table S6).

## Discussion

In plants, 24 nt-siRNAs were found to be the most abundant small RNAs in all kinds of species and tissues studied [23]–[25]. The same trend was found in rice grains, but the inferior grains contained much more 24 nt-siRNAs than superior grains in each of the development stages. As a major component of the small RNA population, siRNAs were speculated to have similar effect of miRNAs in gene regulations [19], [26], [27]. The target gene prediction results indicated that in inferior grains, 24 nt-siRNAs interfered more genes involved in most known rice metabolism pathways, especially in starch and sucrose biosynthesis pathways. These results were consistent with previous studies that poor grain filling of inferior grains was caused by low expression level of starch synthesis genes [5], [7]. It was hypothesized that if the 24 nt-siRNAs could also down regulate expressions of target genes, the 24 nt-siRNAs and their targets should be negatively correlated. However, in the target genes expression data, most of the targets were not negatively correlated with 24 nt-siRNAs. The half-life of targeted mRNAs degradation was quite short as demonstrated in *Arabidopsis* [28]. So, the inconsistency between 24 nt-siRNAs and target genes implicated that 24 nt-siRNAs were not involved in cleavage of target genes.

It was reported that 24 nt-siRNAs bound to Argonaute 4 (AGO4) protein, could direct *de novo* DNA methylation [29], [30]. The asymmetric retention of 24 nt-siRNAs and 24 nt-siRNA*s might be caused by the binding with AGO4, which protect the bound 24 nt-siRNAs from degradation. Conventional views claimed that DNA methylation causing down-regulation of gene expression, but more and more studies showed that gene expression can also be up regulated by DNA methylation [31], [32]. In addition, examples of gene expression activated by dsRNA or RNA-directed DNA methylation were found in both mammals and plants [33]–[35]. Therefore, the higher retention frequency of inferior 24 nt-siRNAs may be a potential reason for higher target gene expression in inferior grains.

The biogenesis of 24 nt-siRNA is delineated as nascent single-stranded RNA (ssRNA) transcription by PolIV, dsRNA synthesis by RDR2, and dsRNA process by DCL3 to form mature 24 nt-siRNAs [21], [36]. Because the synthesis of ssRNA and dsRNA is an energy consuming process [37], more 24 nt-siRNAs in inferior grains need more energy for their synthesis compared with superior grains, which might be a disadvantage for the accumulation of starch during grain filling.

On the other hand, 24 nt-siRNA competes with miRNAs for 2′-OH methylation on the 3′ end of small RNAs. Plant miRNAs and siRNAs carry a 2′-O-methyl group on the 3′-terminal nucleotide catalyzed by HEN1 to increase the stability [38], [39]. As the common substrates, both siRNAs and miRNAs are methylated by HEN1. In *Arabidopsis*, miRNAs methylation in *hen1* partial loss of function lines was rescued by *rdr2* mutant, which indicated that reduction of 24 nt-siRNAs could increase the methylation chance of miRNAs in shortage of HEN1 [24]. This competition might also occur in rice grains, given the fact that inferior grains contained fewer miRNAs than superior grains in each period we sampled (Table S7). In contract, the 24 nt-siRNAs were higher in all the inferior grain samples. Higher frequency of 24 nt-siRNAs may reduce the methylation chances of miRNAs, resulting in a faster degradation rate of miRNAs in inferior grains, which is consistent with the previous conclusions that miRNAs play crucial roles in regulation of genes during grain filling.

### Conclusions

24 nt-siRNAs are the most abundant small interfering RNAs in rice grains aside from microRNAs. In rice superior and inferior grains, the amounts of 24 nt-siRNAs declined with the process of grain filling in both types of grains, but the amount of 24 nt-siRNAs in inferior grains was much higher than that of the superior grains. Bioinformatics prediction indicated that 24 nt-siRNAs targeted on more genes involved in most of the known KEGG rice pathways, such as the starch and sucrose biosynthesis pathway. But combined with digital gene expression profiling of target genes, the majority of 24 nt-siRNAs did not show negative correlations with their corresponding target genes, which might be caused by gene expression activation of DNA methylation. The potential biological meanings for the inconsistency were probably the results of methylation directed gene expression activation, or competition for small RNA stability methylation.

## Materials and Methods

### Plant Materials and Sample Treatments

*Oryza sativa* spp. *japonica* cv. Xinfeng2 was planted in field. Superior and inferior grains were sampled at 10, 15, 21, 27, and 35 days after fertilization. The sample treatments and small RNA isolation and sequencing were conducted essentially according to Peng *et al.* (2011). The same batches of sample RNAs were also subjected to digital gene expression profile sequencing to determine the expression of genes during grain filling in order to study the relationship between small RNAs and their target genes.

### Data Analyses

The small RNA raw data was processed to remove adaptors, low quality tags as well as contaminants to get clean reads. The resulting clean reads sequences were aligned to the rice genome (RGAP, Rice Genome Annotation Project, http://rice.plantbiology.msu.edu/, release 6.1) by using SOAP [40]. siRNAs were identified according to the rule of siRNA duplex structure, which had a perfect matched double strands with two base over-hanging at 3′ end in each single strand [41]. The siRNA reads from each sample were normalized to transcripts per million (TPM) for comparisons according to the following normalization formula: TPM = (siRNA reads/total reads)*1,000,000. Localization of the siRNAs on exon, intron, UTR, repeats, or intergenic region was accomplished using BLAST [42] by comparing against rice database in RGAP (release 6.1).

The digital gene expression profiling raw data was also processed as that of small RNA data to remove adaptors, low quality tags as well as contaminants to get clean reads. All the clean reads were compared with rice cDNA database and only unambiguous tags were used to determine the number of clean tags on each gene. In consideration of SNPs between genotypes, one mismatch was allowed in the tag matching. The number of clean tags of each gene in each library were normalized to TPM as mentioned above.

The potential siRNA target genes were predicted using the online psRNA-Target program (http://bioinfo3.noble.org/psRNATarget/) [43] with default parameters. The predicted target genes were subjected to KEGG analysis (http://www.genome.ad.jp/tools/kaas/) [44] to map genes onto known rice pathways. Correlation between small RNA and target gene expression in superior and inferior grains was evaluated by Pearson correlation index.

### Quantitative Real-time RT-PCR (Q-PCR) Validation of Sequencing Data

Validation of small RNA and digital gene expression data were performed by using Q-PCR as we used previously [18]. Total RNA (1 µg) was reverse-transcribed using a reverse transcriptase enzyme (Promega) and miRNA-specific stem-loop reverse transcription primer as described in [45]. In the Q-PCR test tube, a 5 µl aliquot of 1∶20 diluted cDNA was used as the template in a 20 µl PCR reaction system. The reaction were carried out using SYBR green reaction mix (GoTaq® qPCR Master Mix; Promega) in a BioRad iQ5 sequence detection system (BioRad, USA). The PCR parameters were as following, a pre-incubation at 95°C for 3 min, followed by 40 cycles of denaturation at 95°C for 15 s, annealing at 60°C for 15 s, and extension at 72°C for 32 s. The actin gene was taken as the control gene. The relative fold expression changes of target genes were calculated using the 2 delta-delta Ct method. All gene specific primers used in the experiments are listed in Table S8.

## Supporting Information

Figure S1 **Base frequency of 24 nt-siRNA at each position.** The horizontal axis represents the position of 24 nt-siRNA and the vertical axis represents base frequency.
(TIF)

Figure S2 **Validation of siRNA sequencing data by using quatitative stem-loop RT-PCR. A, C, E, G showed the small RNA sequencing results of four siRNAs in superior and inferior grains in five periods libraries.** B, D, F, H showed the Q-PCR results of the corresponding siRNAs in each libraries.
(TIF)

Figure S3 **Validation of RNAseq data by using quatitative RT-PCR. A, C, E, G, I showed the RNAseq results of genes in superior and inferior grains in five periods libraries.** B, D, F, H, J showed the Q-PCR results of the corresponding genes in each libraries.
(TIF)

Figure S4 **Relative expression level of rice (Nipponbare) DCL3a and DCL3b during the whole life periods.** The horizontal axe indicates the development stages and the vertical axe indicates relative expression level from RNAseq data. The data used was downloaded from Rice Genome Annotation Project database (http://rice.plantbiology.msu.edu/expression.shtml).
(TIF)

Table S1 Frequency of 24 nt-siRNAs greater than 10TPM and their corresponding 24 nt-siRNA*s in each library.
(XLS)

Table S2 **Collection of 24 nt-siRNAs greater than 10TPM and their dynamics in the ten sample libraries during grain filling.**
(XLS)

Table S3 **Expression level of key enzymes involved in producing 24 nt-siRNAs detected by digital gene expression profiling.**
(XLS)

Table S4 **Number of predicted target genes in known rice pathways revealed by KEGG analysis.** Statistic was performed using pair-wise T test between each period of superior and inferior grains.
(XLS)

Table S5 **Frequency of 24 nt-siRNAs linked with expression of corresponding target genes.** The correlation between 24 nt-siRNA and targets was evaluated by Pearson correlation index, index greater 0.878 was significant at the 0.05 level.
(XLS)

Table S6 **Frequency of 24 nt-siRNAs that matched perfectly on the antisense strand of coding exons and the expression level of their corresponding target genes.** The correlation between 24 nt-siRNA and targets was evaluated by Pearson correlation index, index greater 0.878 was significant at the 0.05 level.
(XLS)

Table S7 **Total number counts (in TPM) of siRNAs and miRNAs in each sample libraries.**
(XLS)

Table S8 **Primer sequences used in the Q-PCR analysis.**
(XLS)
