# Supplementary figures and images for: Genome-Wide Analysis of 24-nt siRNAs Dynamic Variations during Rice Superior and Inferior Grain Filling
Source: PLoS One. 2013 Apr 12;8(4):e61029. doi: 10.1371/journal.pone.0061029 (PMC3625182; doi:10.1371/journal.pone.0061029)

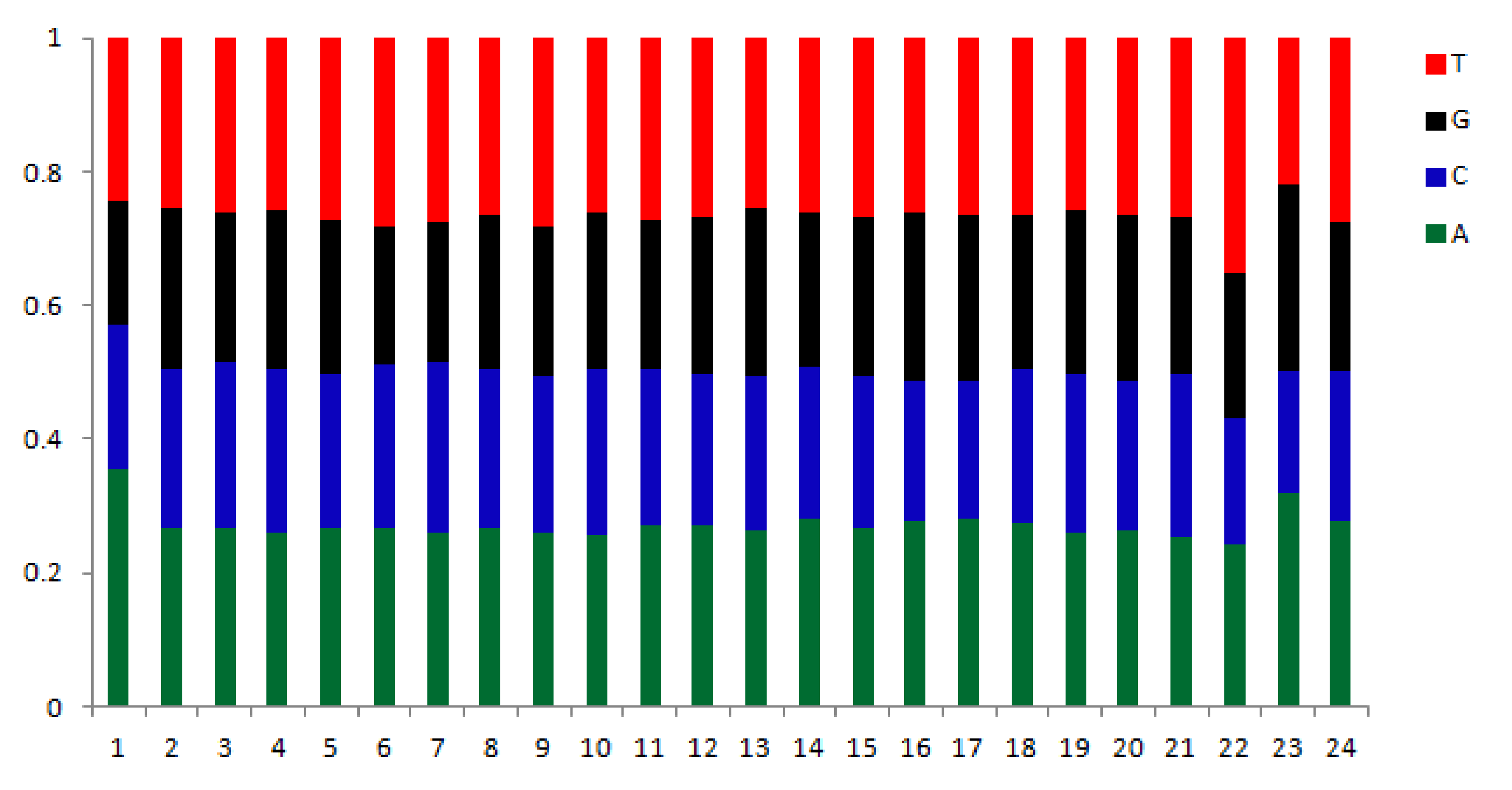

Supplement: Figure S1 — Base frequency of 24 nt-siRNA at each position. The horizontal axis represents the position of 24 nt-siRNA and the vertical axis represents base frequency. (TIF) [file pone.0061029.s001.tif]

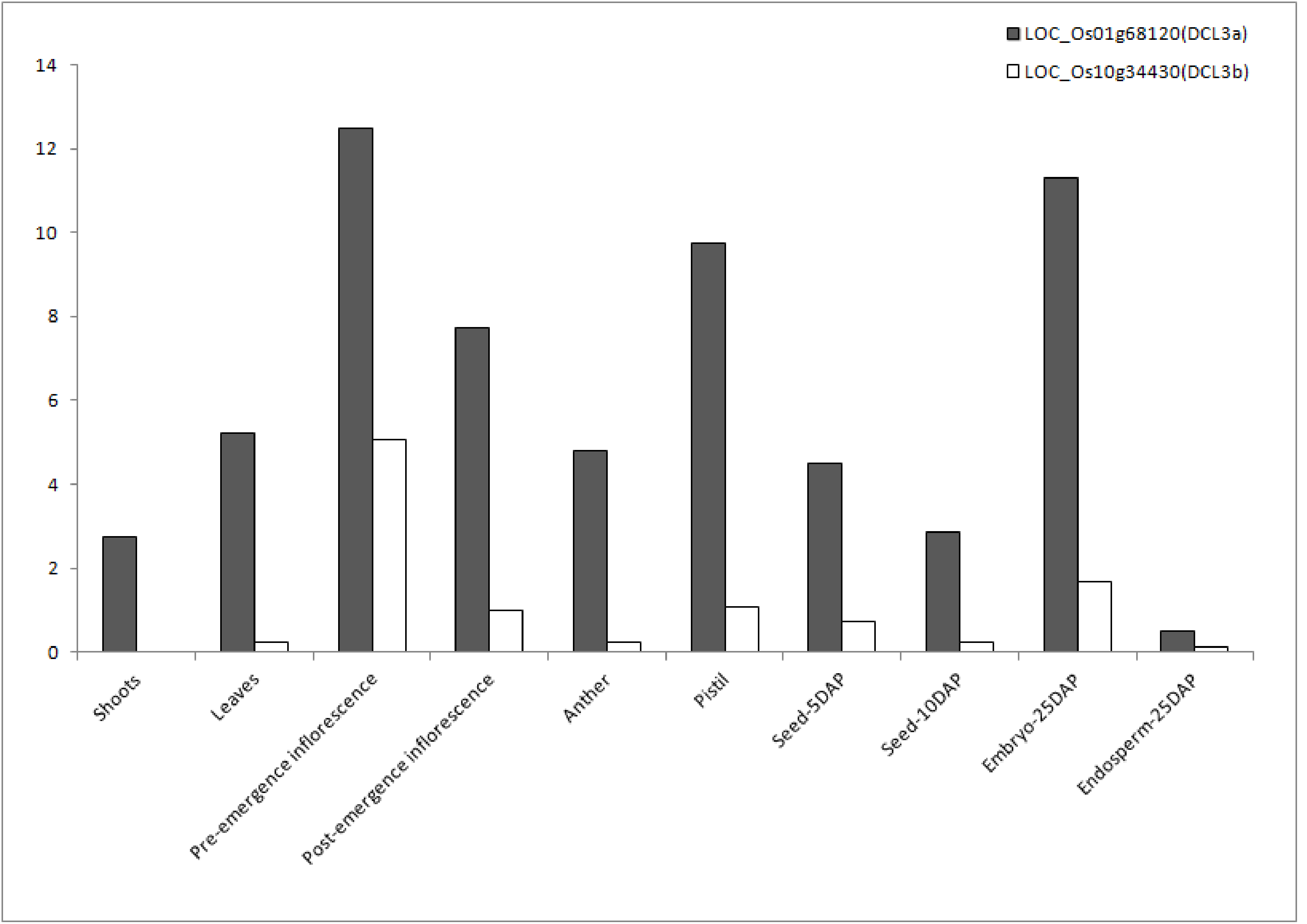

Supplement: Figure S4 — Relative expression level of rice (Nipponbare) DCL3a and DCL3b during the whole life periods. The horizontal axe indicates the development stages and the vertical axe indicates relative expression level from RNAseq data. The data used was downloaded from Rice Genome Annotation Project database (http://rice.plantbiology.msu.edu/expression.shtml). (TIF) [file pone.0061029.s004.tif]
